# Incorporating *a-priori* information in deep learning models for quantitative susceptibility mapping via adaptive convolution

**DOI:** 10.3389/fnins.2024.1366165

**Published:** 2024-03-11

**Authors:** Simon Graf, Walter A. Wohlgemuth, Andreas Deistung

**Affiliations:** ^1^University Clinic and Polyclinic for Radiology, University Hospital Halle (Saale), Halle, Germany; ^2^Halle MR Imaging Core Facility, Medical Faculty, Martin-Luther-University Halle-Wittenberg, Halle, Germany

**Keywords:** quantitative susceptibility mapping, magnetic resonance imaging, deep learning, adaptive convolution, transfer learning, *a-priori* information

## Abstract

Quantitative susceptibility mapping (QSM) has attracted considerable interest for tissue characterization (e.g., iron and calcium accumulation, myelination, venous vasculature) in the human brain and relies on extensive data processing of gradient-echo MRI phase images. While deep learning-based field-to-susceptibility inversion has shown great potential, the acquisition parameters applied in clinical settings such as image resolution or image orientation with respect to the magnetic field have not been fully accounted for. Furthermore, the lack of comprehensive training data covering a wide range of acquisition parameters further limits the current QSM deep learning approaches. Here, we propose the integration of a priori information of imaging parameters into convolutional neural networks with our approach, adaptive convolution, that learns the mapping between the additional presented information (acquisition parameters) and the changes in the phase images associated with these varying acquisition parameters. By associating *a-priori* information with the network parameters itself, the optimal set of convolution weights is selected based on data-specific attributes, leading to generalizability towards changes in acquisition parameters. Moreover, we demonstrate the feasibility of pre-training on synthetic data and transfer learning to clinical brain data to achieve substantial improvements in the computation of susceptibility maps. The adaptive convolution 3D U-Net demonstrated generalizability in acquisition parameters on synthetic and in-vivo data and outperformed models lacking adaptive convolution or transfer learning. Further experiments demonstrate the impact of the side information on the adaptive model and assessed susceptibility map computation on simulated pathologic data sets and measured phase data.

## 1 Introduction

Quantitative imaging of specific physical properties using magnetic resonance imaging (MRI) is of tremendous interest as these images are expected to be directly comparable across imaging sites and time points and directly link to the underlying tissue substructure (e.g., myelination, iron, anisotropy) (Deistung et al., 2013; Weiskopf et al., 2021). Apart from non-invasive mapping of relaxation times like T1, T2 and T2* or diffusion-based parameters, characterization of the magnetic susceptibility distribution using MRI, commonly referred to as quantitative susceptibility mapping (QSM), has attracted considerable interest, particularly for characterizing iron and calcium accumulations (Schweser et al., 2010), myelination (Li et al., 2011) and venous vasculature (Fan et al., 2014; Ward et al., 2018) in the human brain. Thus, the specific information that can be evaluated with QSM is useful for many neurological and psychological applications (Fushimi et al., 2023). In QSM, the susceptibility distribution is deduced via sophisticated processing from raw gradient-recalled echo phase images commonly considering the corresponding magnitude images as source for prior information. The usual QSM post-processing stream includes multi-channel coil combination (usually provided by the MRI scanner), field map estimation and background field removal to provide the local magnetic field (i.e., the magnetic field resulting from the tissue of interest such as brain tissue). This data is finally converted via field-to-susceptibility inversion into magnetic susceptibility maps (Deistung et al., 2017). The field-to-susceptibility inversion is an ill-posed problem that cannot be solved directly, making the use of regularization or deep learning approaches necessary, and for which a variety of approaches have been suggested (Bilgic et al., 2021). While in the last decade mainly regularization approaches have been employed (Langkammer et al., 2018), throughout the last years dipole inversion approaches relying on deep learning (DL) have become popular due to their non-linear mapping capabilities and computational efficiency (Jung et al., 2020a). In their implementation (QSMnet), Yoon et al. (2018) applied a 3D U-Net (Ronneberger et al., 2015) for field-to-susceptibility inversion trained with *in vivo* susceptibility maps calculated based on multiple measurements with different head orientations during the MRI (Liu et al., 2009), which was further enhanced by utilizing extensive data augmentation (QSMnet+) (Jung et al., 2020b). With DeepQSM (Bollmann et al., 2019), a similar approach, but trained on purely synthetic data consisting of simple predefined geometric shapes, it could be shown by its application to *in vivo* brain data that the model was able to learn the underlying physics. Further proposed DL-approaches were xQSM (Gao et al., 2020), QSMGAN (Chen et al., 2020), autoQSM (Wei et al., 2019), and iQSM (Gao et al., 2022). Recently, unrolled models have also been suggested to solve the field-to-susceptibility problem (Lai et al., 2020; Polak et al., 2020; Feng et al., 2021). All approaches mentioned so far lack on generalizability as they assume isotropic voxel-sizes and purely axial orientation preventing their use on real-life gradient-echo data with anisotropic voxel sizes or oblique field-of-view (FoV) orientation, a usual scenario in daily clinical routine. While the supervised Meta-QSM approach (Liu and Koch, 2019) and the unsupervised resolution agnostic AdaIn-QSM approach (Oh et al., 2022) addressed the aspect of different image resolutions, the FoV orientation was still assumed purely axial. Just very recently, Xiong et al. (2023) introduced AFTER-QSM that accounts for oblique FoV orientation and anisotropic voxels in field-to-susceptibility inversion by employing affine transformations into a purely axial coordinate space with isotropic resolution. The U-Net for inversion is applied in axial space and the resulting susceptibility maps are then retransformed into the native coordinate space. Finally, a super-resolution network consisting of residual dense blocks is applied to the retransformed susceptibility map to overcome blurring due to the spatial transforms. AFTER-QSM, however, suffers from over-sharpening and increased noise due to the super-resolution network, reducing its use in clinical brain imaging. So far, the suggested DL-based QSM inversion approaches considered only the acquired image data, neglecting easily available additional *a-priori* information (e.g., acquired voxel size, field-of-view orientation to the static main magnetic field) that is required in conventional regularization-based field-to-susceptibility approaches.

Challenges in current deep learning approaches, not only in QSM, are data scarcity, the lack of large-scale and comprehensive training data, to learn internal representations that lead to comprehensive invariance against changes in data. In gradient-echo MRI, the choice of imaging parameters (e.g., voxel size, FoV orientation) substantially affect the local magnetic field representation and field direction in the acquired volume. However, these parameters are known and available *a-priori* and could be incorporated into field-to-susceptibility inversion.

Inclusion of such *a priori* information is not just an issue in the field of QSM but occurs in other use cases like crowd counting as well. Including this additional information directly into the network model and network training however is non-trivial. One simple approach is the inclusion via an extra image channel storing the additional information similar to gray value and RGB-channels. Kang et al. (2020) proposed the direct manipulation of network parameters by including geometric dependencies in crowd counting and leverage information about the camera angle.

Within this work, we propose a new and effective approach, motivated from crowd counting with neural networks referred to as adaptive convolution, to solve the field-to-susceptibility inversion in QSM for gradient-echo data acquired with arbitrary voxel dimensions and image orientations, without the need of applying affine image transformations and additional super resolution techniques. With this approach we demonstrate the integration of these data acquisition properties into the network model for the first time by providing known auxiliary information to the network model as opposed to learning these dependencies solely from the image data itself. Finally, the feasibility and applicability of network pre-training on synthetic in-silico data followed by transfer learning toward *in vivo* MRI data is presented, thus avoiding the risk of an anatomical bias in the deep learning model.

## 2 Methods

### 2.1 Ill-posed dipole inversion problem of QSM

The magnetic field perturbation ${\vec{B}}_{M}$($\vec{r}$) measured with MRI, which is caused by the tissue magnetization of the underlying magnetic susceptibility distribution $\chi \left(\vec{r}\right)$ of the biological tissue, is defined in Eqs. 1a–1c according to Marques and Bowtell (2005)


(1a) $$\begin{array}{l} \vec{B_{M}}\left(\vec{r}\right)=\frac{\partial }{\partial \vec{r}}\times \frac{\mu_{0}}{4\pi }∭d^{3}\vec{r}’\;\vec{M}\left(\vec{r}’\right)\times \frac{\partial }{\partial \vec{r}}\frac{1}{\left|\vec{r}-\vec{r}’\right|} \end{array}$$

(1b) $$=\frac{\mu_{0}}{4\pi }∭d^{3}\vec{r}’\;\frac{\partial }{\partial \vec{r}}\times \frac{\vec{M}\left(\vec{r}’\right)\times \left(\vec{r}-\vec{r}’\right)}{{\left|\vec{r}-\vec{r}’\right|}^{3}}$$

(1c) $$=\frac{\mu_{0}}{4\pi }∭d^{3}\vec{r}’\;\left(\frac{3\vec{M}\left(\vec{r}’\right)\left(\vec{r}-\vec{r}’\right)}{{\left(\vec{r}-\vec{r}’\right)}^{5}}\cdot \left(\vec{r}-\vec{r}’\right)-\frac{\vec{M}\left(\vec{r}’\right)}{{\left(\vec{r}-\vec{r}’\right)}^{3}}\right),$$

where $\vec{M}\left(\vec{r}\right)$ is the magnetization $\mu_{0}$ the magnetic field constant and $\vec{r}$ the coordinate position in image space. Eq. 1c can be expressed as a convolution between the magnetic field perturbation ${\vec{B}}_{M}\left(\vec{r}\right)$ and the point dipole response $d\left(\vec{r}\right)$ (Li and Leigh, 2004; Marques and Bowtell, 2005):


(2) $$d\left(\vec{r}\right)=\frac{1}{4\pi }\cdot \frac{3\cos^{2}\left(\theta \right)-1}{{\left|\vec{r}\right|}^{3}}=F^{-1}\left(\frac{1}{3}-\frac{k_{zp}^{2}}{{\left|\vec{k}\right|}^{2}}\right),$$

where $\vec{k}$ are the coordinates in k-space, $k_{zp}$ the object coordinates projection onto the main magnetic field that is assumed to align along z-direction and θ is the angle between the z-direction and $\vec{r}$ (Liu et al., 2009). Assuming first order approximation for non-ferromagnetic materials $\left(\left|\chi \right|\ll 1\right)$, separating near- and far-field contributions via the Lorentz sphere (Schweser et al., 2016) and employing the relationship between the susceptibility distribution and the resulting magnetic field variations (Li and Leigh, 2004; Marques and Bowtell, 2005), the relationship between the magnetic susceptibility distribution and the magnetic field perturbation is given by Eq. 3.


(3) $$\vec{B_{M}}\left(\vec{r}\right)=\vec{B_{0}}\int d^{3}\vec{r^{\prime }}\chi \left(\vec{r^{\prime }}\right)\cdot d\left(\vec{r}-\vec{r^{\prime }}\right)=F^{-1}\left\{\chi \left(\vec{k}\right)\cdot d\left(\vec{k}\right)\right\}\vec{B_{0}}$$

Since the dipole kernel $d\left(\vec{k}\right)$ has zeros at the magic angle (θ≈54.7°) and low values in the vicinity of the conical surface, this relationship is ill-posed, posing a challenge in calculating the susceptibility distribution. Therefore, algorithms including proper regularization techniques, such as MEDI (Liu et al., 2012) or HEIDI (Schweser et al., 2012), are required to determine susceptibility maps from a single MRI scan. Alternatively, the approach ‘calculation of susceptibility through multiple orientation sampling’ (COSMOS) (Liu et al., 2009) relies on at least three MRI scans with varying orientations of the object (e.g., the head) to the main magnetic field to shift the undetermined k-space regions and comprehensively fill the entire k-space. Based on Eqs 2, 3 it becomes obvious that the image resolution as well as the orientation of the FoV with respect to the main magnetic field impacts the magnetic field perturbation, where tilting the FoV leads to a change in the alignment of the FoV axes with physical axes describing the main magnetic field. For in-silico experiments and generation of training data, tilting of the FoV was mimicked by tilting the main magnetic field direction.

### 2.2 Adaptive convolution

**Figure 1:**
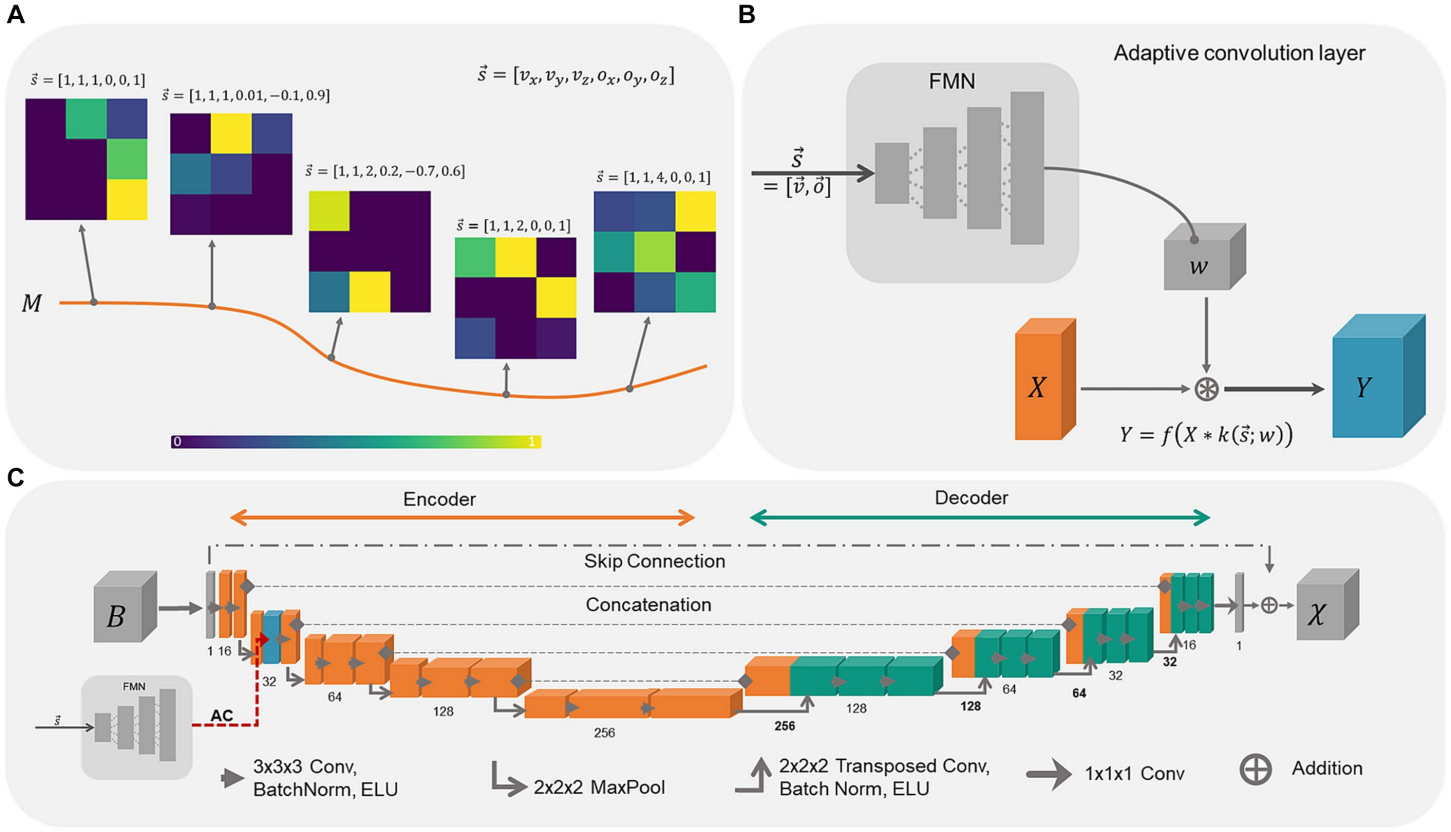
Schematic overview of adaptive convolution, adaptive layers and the used 3D U-Net architecture. **(A)** The filter manifold compresses the relationship between the side information $\vec{s}$ and the changes in the image onto a low dimensional filter manifold in the high dimensional filter weight space. By changing the side information, the filter kernel values itself change, sweeping along the smooth filter manifold. **(B)** Adaptive convolutional layers are built from the Filter Manifold Network (FMN) consisting of 4 fully connected linear layers that compute the weights *w* of the respective convolution operation of the input feature maps *X*, yielding the output feature maps *Y* (blue block). **(C)** The 3D U-Net is composed of an encoder (orange blocks) and a decoder (turquoise blocks) with the adaptive convolution layer (dashed red arrow) included in the first encoding stage (blue block).

The concept behind adaptive convolution is the selection of the most appropriate set of convolutional filter weights for specific data sets, by providing additional information (Kang et al., 2020). When the convolutional filter weights are considered as points on a low-dimensional manifold in the high-dimensional filter weight space, the weights move on the manifold as a function of the additional information (Figure 1A). Hence, the convolution filter weights change adaptively as a function of the presented side information. The processing of this additional information as well as the computation of convolutional filter weights is performed by the filter manifold network (FMN), a fully connected feed forward network. When using a tilted FoV and/or anisotropic voxel sizes for MRI data acquisition, the shapes and edges of the anatomical structures in the image change smoothly. Consequently, by correlating these changes in the image with the additional information provided via the FMN, the network model is expected to learn the relationship between the susceptibility map and B-field distribution more easily. While the filter manifold is learned by the FMN during training, in inference the FMN weights remain fixed, however, the corresponding weights of the convolution operation change adaptively. Adaptive convolution is employed to adjust network parameters to data-specific attributes, selecting appropriate parameters for the susceptibility map calculation.

Adaptive convolution layers are built from fully connected feed forward networks as shown in Figure 1B. The FMN receives the side information $\vec{s}$ consisting of six input parameters, i.e., voxel-size $\vec{v}$ and the image orientation $\vec{o}$, at the input layer. The information propagates through the linear layers with ELU activation function *f*, whereby the dimensions of the extracted feature maps of the hidden layers are gradually increased (12, 48, 196). The number of extracted features at the final layer depends on the placement of the adaptive layer in the network to match the size of the corresponding convolution kernel. Since we placed the adaptive convolution layer in the first encoding block, the final FMN layer encodes 13,824 parameters that are reshaped into the 5D-tensor with shape [16, 32, 3, 3, 3] encoding the filter weights *w.* Thereafter, the input feature map *X* from the previous convolution layer is convolved with the adaptive convolutional filter *k* yielding the output feature map *Y*. The adaptive layer weights, and consequently the weights of the FMN, are updated and adjusted during network training. This enables the FMN to associate the side information with the filter manifold.

### 2.3 Adaptive U-Net architecture

The adaptive U-Net (Figure 1C) computes susceptibility maps from the same-sized local magnetic field perturbation ($\vec{B_{M}}\left(\vec{r}\right)$). The network relies on a standard 3D U-Net (Ronneberger et al., 2015) with 16 initial channels, concatenations between encoder and decoder and batch normalization layers (Ioffe and Szegedy, 2015). A skip connection between input and output was included to enable residual learning (Kim et al., 2016), which increases the performance of the network (Jin et al., 2017) and helps to reduce vanishing gradients during training (He et al., 2016). Thus, the network model computes the difference between the magnetic field perturbation and the susceptibility map. The ELU activation function (Clevert et al., 2015) was used to further address vanishing gradients and due to its greater ability to generalize. The adaptive convolution layer was included in the first encoding block. The kernel sizes of the convolutional layers were (3x3x3) and for transposed convolution (2x2x2). As comparison, an adaptive U-Net with the adaptive layer included in the entire encoder (Supplementary Figure S1), in total 10 adaptive convolution layers, and an identical U-Net lacking adaptive convolution were constructed and trained similarly as well as evaluated against the adaptive U-Net. This adaptive U-Net is referred to as adaptive encoder U-Net.

### 2.4 Model-based training/loss function

The loss measure (Eq. 4) used for training the models was designed as such that the similarity of the susceptibility maps and magnetic field perturbations is measured, including model-based learning therewith. $L_{\chi }$ compares the ground truth susceptibility $\chi_{gt}$ with the reconstructed susceptibility map $\chi_{rec}\text{.}$ $L_{B}$ compares the ground truth magnetic field $B_{gt}$ with the one computed via the forward model (Eq. 3), where the reconstructed susceptibility map $\chi_{rec}$ was convolved with the unit dipole response $d_{\vec{v},\vec{o}}$ given by the specific voxel-size $\vec{v}$ and image orientation $\vec{o}$. The inclusion of the forward field computation ensures that optimization occurs only towards solutions obeying this convolutional relationship. The weighing factor λ was set to λ=1, to ensure that the physical model is substantially enforced via the loss function.


(4) $$L=L_{\chi }+\lambda L_{B}=∥\chi_{gt}-\chi_{rec}{∥}_{2}^{2}+\lambda ∥B_{gt}-\left(\chi_{rec}⊛d_{\vec{v},\vec{o}}\right){∥}_{2}^{2}$$

### 2.5 Network training

To circumvent data sparsity of brain MRI data with multiple head orientations, voxel sizes and ground truth susceptibility maps, and consequently allow the network model to learn solving the underlying physical problem of field-to-susceptibility inversion, pre-training on synthetic data sets and transfer learning to *in-vivo* brain data was performed. Hence, the network models were pre-trained on 1,000 purely synthetic data sets for 500 epochs, detaching the model from developing a possible anatomic bias of brain data therewith. The number of data sets was chosen empirically as trade-off between using a sufficient amount of training data and network training time. The synthetic susceptibility maps (shape: 320 × 320 × 320 voxel) were composed of rectangles, polygons and ellipsoids with susceptibilities drawn from a Gaussian distribution $N\left(\mu =0,\sigma =0.25\right)$. The total number of shapes was randomly drawn, ranging from 80 to 150 rectangles and 200 to 300 ellipsoids. The number of polygons was set to 50 empirically. 300 ellipsoids of random dimension were used for the creation of the mask and randomly assembled in image border regions. Each shape was blurred individually with a Gaussian kernel from the normal distribution $N\left(\mu =0,\sigma =U\left(0,0.8\right)\right)$ to smooth the edges. σ was drawn from a uniform distribution U. The susceptibilities of overlaying shapes were averaged to increase the number of edges in the data sets. The magnetic field perturbation was obtained via fast forward convolution in k-space (Eq. 3). During network training, four patches of dimension 160 × 160 × 160 were randomly cut from each data set. Augmentation techniques included random scaling of the susceptibilities, addition of Gaussian noise, random flipping and random 90° rotations for the patches. To obtain magnetic field distributions of random voxel size and image orientations, the dipole kernel for computing the field distributions and the ground truth susceptibility maps were augmented. The voxel-size was randomly drawn from the Gaussian distribution $N\left(\mu =1,\sigma =1.5\right)$. Different FoV orientations were simulated by tilting the main field direction of the dipole kernel. The normalized field vector ${\vec{B}}_{0}={\left[0,0,1\right]}^{T}$ was randomly rotated around the x-, y- and z-axis. The x- and y-values were drawn from the Gaussian distribution $N\left(\mu =0,\sigma =11\right)$ and the z-values from $N\left(\mu =0,\sigma =15\right)$. Variations in voxel-size and image orientation occurred with probability 0.8, ensuring standard parameters in the training data, and were used to generate the dipole kernel for the forward convolution of susceptibility maps.

In a second step, the network models were tailored specifically to brain data by performing transfer learning to susceptibility maps reconstructed from multi-orientation brain data sets acquired at 3 T (Shi et al., 2022) and 7 T (Deistung et al., 2013). The training data consisted in total of 109 data sets of varying orientations. Since the data sets were originally acquired with isotropic voxel sizes, we applied trilinear interpolation to augment the data sets to anisotropic voxel sizes. The voxel-size was randomly drawn from the distribution $N\left(\mu =1,\sigma =1.5\right)$. During transfer learning, it was randomly selected with a probability of 0.5 whether the measured local magnetic field perturbation or whether the magnetic field computed by fast forward convolution of the ground truth COSMOS susceptibility map with the dipole kernel was used as input data. In the latter case, the susceptibilities of the COSMOS maps were scaled by applying a randomly chosen multiplication factors (interval [0, 2]) to further augment the training data. By performing random scaling throughout transfer learning, the network model not only optimizes toward the susceptibility distribution of the provided data, but also maintains the susceptibility scale invariance. Four patches with dimensions of 96 × 96 × 96 were randomly cut from the data sets and padded to 128 × 128 × 128 to ensure border regions in the data. Transfer learning was performed for 30 epochs.

The AdamW optimizer $\left(\beta_{1}=0.9,\beta_{2}=0.99,\varepsilon =1e-08,\lambda =0.01\right)$ (Loshchilov and Hutter, 2017) was used during training and transfer learning. A cosine annealing learning rate schedule with warm restarts ($T_{0}=500,T_{\text{mult}}=0.5,\eta_{\text{init}}=0.001,\eta_{\text{min}}=1e-08$) was used for network pre-training (Loshchilov and Hutter, 2016). For the transfer learning, the parameters of the adaptive layer remained fixed, the learning rate for all parameters in the decoding branch of the model was set to η=0.001 and to η=0.00001 for all other parameters. All augmentations, interpolations and dipole convolutions were performed on the GPU. Automated mixed precision together with cuDNN-benchmarking was used during distributed training of the network models on four NVIDIA A100 GPUs (NVIDIA Corporation, Santa Clara, CA, United States). It took approximately 25 h to train the model for 500 epochs and additional 3 h for transfer learning. Training the adaptive U-Net with four patches of voxel-size 160 × 160 × 160 requires around 26 GB of GPU memory per used GPU. The application of the trained adaptive U-Net to a local field map with a dimension of 192 × 224 × 160 voxels requires 18 GB CPU memory and takes approximately 14.3 s on a AMD EPYC 7713 CPU (Advanced Micro Devices Corporation, Santa Clara, CA, United States) or 1.6 s on a NVIDIA A100 GPU.

### 2.6 Evaluation data

The adaptive U-Net and the conventional U-Net without and with transfer learning were evaluated on synthetic data sets as well as *in vivo* brain data sets (Table 1). Gradient-echo data were acquired on a Siemens Magnetom Vida (Siemens Healthcare GmbH, Erlangen, Germany) with a 64-channel head coil. One healthy subject was scanned three times. Two measurements were conducted with the isotropic voxel size of 1 mm × 1 mm × 1 mm, but different FoV orientations, one was oriented purely axial ($\vec{o}$ = [0, 0, 1]^T^) and one was tilted by 12-degree from the axial orientation ($\vec{o}=[$–0.15, −0.20, 0.97]^T^) (Table 1, this study (1)). A third scan with 0.57 mm × 0.57 mm × 2 mm voxels and a FoV tilted by 25° from the axial plane ($\vec{o}=[$–0.03, –0.42, 0.90]^T^, Table 1, this study (2)) was acquired to test the limits of the network models. Since the side information array consisted of voxel sizes $v_{i}\geq 1$, this data set serves to assess the network model performance on out-of-distribution data.

**Table 1:** Acquisition parameters of *in-vivo* evaluation data.

Acquisition parameters	Deistung 2013	Shi 2022	This study (1)	This study (2)
Echo Time(s) [ms]	10.5	7.7, 13.4, 18.8, 25.3, 31.7, 38.2	7.04, 13.75 20.46, 27,17	7.41, 14.57, 21.73, 28.89
Repetition Time [ms]	17	44	32	35
Flip angle [°]	8	20	15	15
Voxel-size [mm, mm, mm]	[0.4, 0.4, 0.4]	[1, 1, 1]	[1, 1, 1]	[0.57, 0.57, 2]
Magnetic field strength [T]	7	3	3	3
Scanner	Siemens Magnetom 7 T	Siemens Magnetom Prisma	Siemens Magnetom Vida	Siemens Magnetom Vida

Post-processing of the data acquired within this study consisted of unwrapping (Abdul-Rahman et al., 2007) the phase images of each echo, dividing them by $B_{0}*TE_{i}*\gamma$ and averaging them across the various echo times TE to achieve the magnetic field perturbation measured at 1 T. Sophisticated harmonic artefact removal for phase data (Schweser et al., 2011) with 10 different spherical kernels (1 to 10 voxels, regularized with truncated singular value decomposition: 0.1) was applied to reveal the local magnetic field perturbation. These SHARP-processed images were used for field-to-susceptibility inversion with the different deep learning models and homogeneity enabled incremental dipole inversion (HEIDI) (Schweser et al., 2012), while the maps computed using HEIDI served as reference. We referenced all susceptibility maps to the average susceptibility of the brain tissue within the field of view and stated susceptibility values in parts-per-million (ppm).

Data from other studies Deistung et al. (2013); Shi et al. (2022) were also considered for transfer learning and performance evaluations. Scan parameters of these studies are summarized in Table 1. For explicit details on data post-processing the reader is referred to the corresponding articles by Deistung et al. (2013) and Shi et al. (2022).

To evaluate the performance of the adaptive U-Net on pathological data, we simulated a data set containing four arbitrary shaped lesions. To this end, a COSMOS map from Shi et al. (2022) was interpolated to voxel-size 1 mm x 1 mm x 1.5 mm based on which a mask with four lesions was manually drawn. Various susceptibility values ($\chi =\left[-1.0,-0.5,0.5,1.0\right]$) were assigned to the mask regions, smoothed using a Gaussian kernel (μ = 0, σ = 0.5), and incorporated into the COSMOS map. The magnetic field perturbation for the COSMOS data set with incorporated lesions was calculated via fast forward convolution in k-space (Eq. 3).

The reconstructed susceptibility maps of the adaptive U-Net were compared with other field-to-susceptibility methods including the adaptive U-Net without transfer learning, the conventional U-Net with transfer learning, the adaptive encoder U-Net, AFTER-QSM (Xiong et al., 2023) and HEIDI. We downloaded the AFTER-QSM model (implementation and trained network parameters) from the authors GitHub repository (https://github.com/sunhongfu/deepMRI/tree/master/AFTER-QSM, accessed and downloaded on 31.01.2024). Since AFTER-QSM is a DL-based field-to-susceptibility model specifically addressing resolution and orientation invariance and the trained parameters are publicly available, we chose this method for comparison.

Quality assessment of the reconstructed susceptibility maps has been performed on visual inspection in combination with quantitative image metrics. For this, the normalized root mean-squared-error (NRMSE, root mean squared error divided by the L2-norm of the ground truth and multiplied by 100), the structural similarity index (SSIM) and the peak signal-to-noise ratio (PSNR) were employed. To assess the amplitude of the computed susceptibilities, 400 k values were randomly sampled from the computed (c) and ground truth (g) susceptibility maps; ordinary linear least-squares fitting was then applied to determine the functional relationship g(c) (assuming g to be precise values). A mask of positions throughout the whole brain was randomly created and applied to the data sets. Hence, susceptibilities from identical positions were drawn and the random seed was identical for all models. Furthermore, the intensity values of the computed and ground truth susceptibility maps within cortical and deep gray (caudate nucleus, globus pallidus, thalamus, putamen, hippocampus, red nuclei, substantia nigra) matter brain regions were assessed via scatter plots and ordinary least-squares fitting. The gray matter regions were identified using DL-based segmentation (Billot et al., 2023) on the COSMOS map and then reduced in size by applying erosion with a 3 × 3 × 3 matrix of ones as structural element.

## 3 Results

### 3.1 Assessment of adaptive layer activations

**Figure 2:**
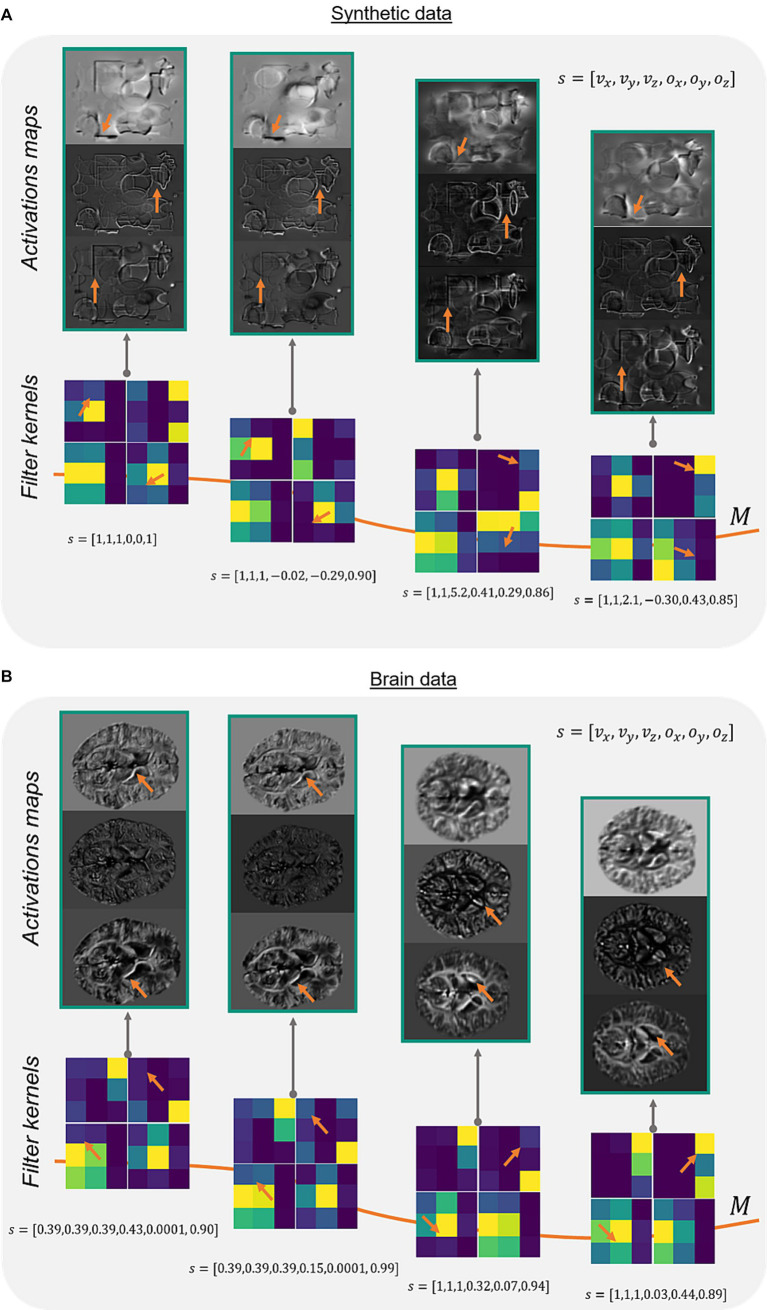
Network activations and filter kernels for different side informations. Three different activation maps of the same image slice on a synthetic data set **(A)** and corresponding image slices on a brain data set **(B)** show varying portions of extracted edges and intensities (orange arrows) depending on different side information s (array of the acquisition parameters voxel-size $\vec{v}$ and FoV orientation $\vec{o}$). The adaptive filter kernels change regarding feature extraction based on the presented side information (orange arrows). Exemplarily, identical network layer activation maps and filter kernels of the adaptive layer (Figure 1C, blue) are shown in **(A,B)** for different input data. M represents the filter manifold.

To verify the method of adaptive convolution itself and check, whether the adaptive layer has learned the filter manifold, different network activation maps and adaptive filter kernels were investigated for synthetic and brain data (Figure 2). Comparing the activation maps reveals changes in intensities and prominent edges in the respective feature maps (Figure 2, orange arrows). This change of structures is dealt in the adaptive model with rotating the filter kernel itself (Figure 2, orange arrows). On synthetic data and brain data, a constant change in edge sensitivity can be noticed by, e.g., changing the orientation of the edges, changing vertical edges to corners, or flipping filter values.

### 3.2 Evaluation on synthetic data

**Figure 3:**
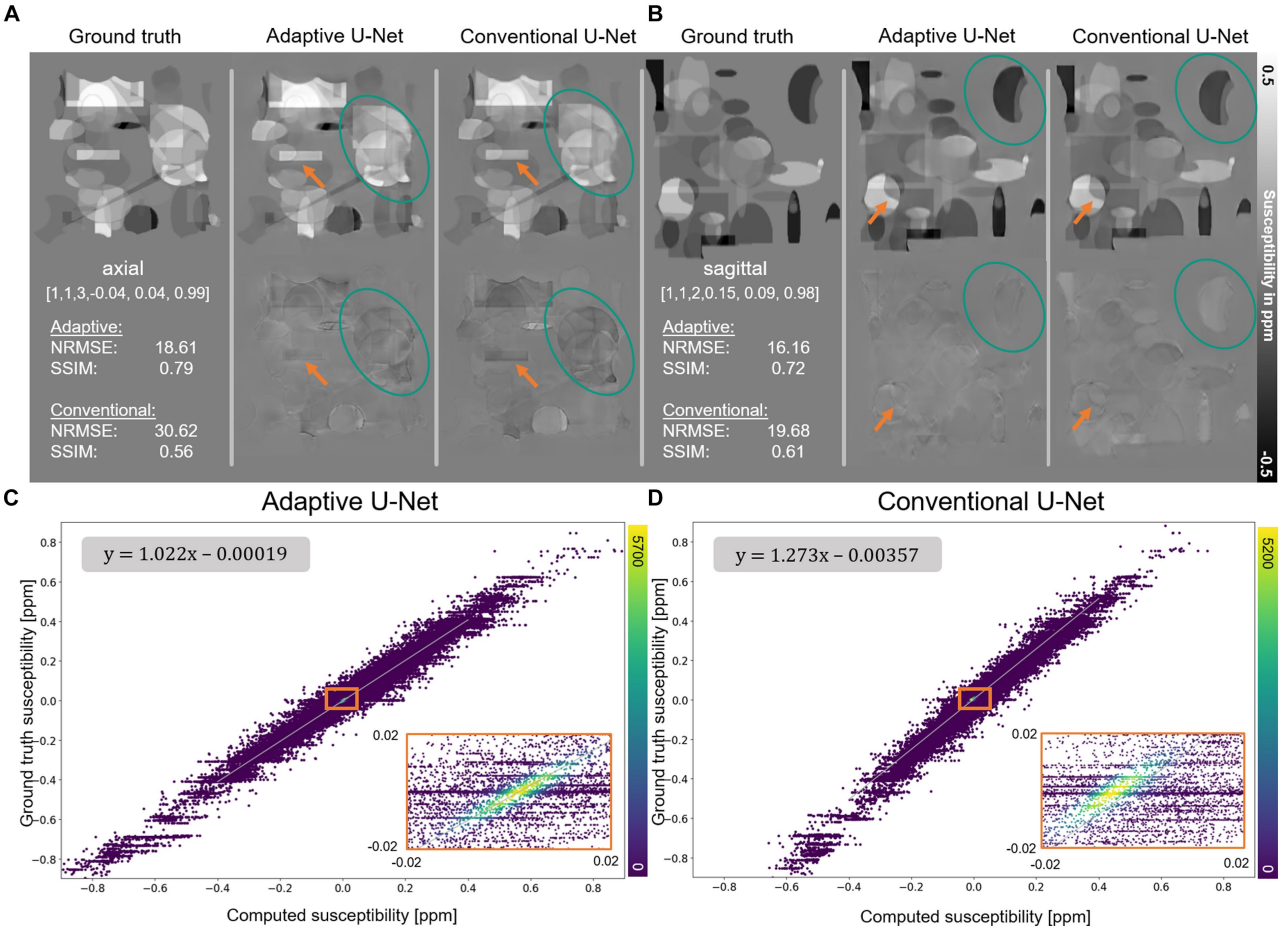
Reconstruction of synthetic susceptibility data. Comparison of computed susceptibility maps from the adaptive U-Net and conventional U-Net on synthetic data sets in **(A)** axial and **(B)** sagittal view. The top row in **(A,B)** shows the ground truth and reconstructed susceptibility maps, whereas the error maps (differences between reconstructed and ground truth susceptibility) are presented in the second row. Variations from ground truth susceptibility maps are observed at the individual objects’ edges (orange arrows) and the susceptibilities (turquoise ellipses) themselves. The normalized root mean squared error (NRMSE) and the structural similarity index (SSIM), depicted in the left inlet in the second row, indicate higher similarity of the susceptibility map reconstructed with the adaptive U-Net to the ground truth. **(C,D)** Scatter plot of 400 k randomly drawn susceptibilities with color encoded density from the data sets shown in **(A)**. In the upper left corner, the functional relationship obtained by linear ordinary least-squares fitting is depicted. Enlarged sections are framed by an orange rectangle.

The computed susceptibility maps of the adaptive 3D U-Net and the conventional 3D U-Net generally resemble the ground truth susceptibility (Figure 3), however, differences in reconstructed object details and susceptibility intensities are observed (orange arrows). Indicated by lower NRMSE and higher SSIM the adaptive model computes susceptibility maps with higher similarity to ground truth data than the conventional U-Net. The plot of the susceptibilities of the adaptive model reconstruction (Figure 3C) against the ground truth susceptibilities shows a point distribution centered on a straight line, having highest point density around zero. The linear fitting showed a slope of 1.022, indicating a high degree of concordance between the calculated and ground truth susceptibility. For the conventional model (Figure 3D), the distribution between computed and ground truth susceptibilities is shifted toward a smaller susceptibility range, which is evident by a slope of 1.273 of the fitting curve.

### 3.3 Evaluation on data similar to the training dataset

**Figure 4:**
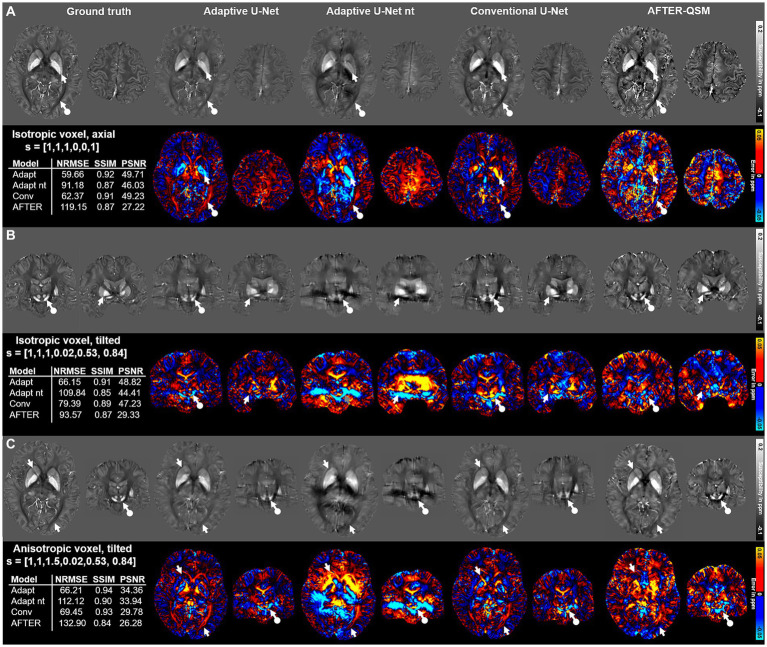
Comparison of the different network models on *in vivo* brain data sets with different acquisition parameters provided by Shi et al. (2022). Susceptibility maps were computed for three data sets by the adaptive U-Net with transfer learning (Adaptive U-Net), the adaptive U-Net without transfer learning (Adaptive U-Net nt), the conventional U-Net with transfer learning and the AFTER-QSM approach (Xiong et al., 2023). The different QSM approaches were evaluated on a dataset with isotropic voxel size (1 mm × 1 mm × 1 mm) and pure axial acquisition ($\vec{o}=$[0, 0, 1]^T^) **(A)**, a dataset with isotropic voxel size (1 mm × 1 mm × 1 mm) and a tilted FoV ($\vec{o}=$[0.02, 0.53, 0.84]^T^) **(B)** and a dataset with anisotropic voxel size (1 mm x 1 mm x 1.5 mm) obtained by trilinear interpolation and a tilted FoV $(\vec{o}=$[0.02, 0.53, 0.84]^T^) **(C)**. The rows with gray background show the computed susceptibility maps, the rows with black background the respective difference maps to the ground truth. Arrows highlight prominent differences in the computed susceptibility maps. The normalized root mean squared error (NRMSE), the structural similarity index (SSIM) and the peak signal-to-noise ratio (PSNR) serve as quantitative image metrics and are presented left. Transfer learning was performed on similar data from Shi et al. (2022).

The computed susceptibility maps of the different network models differ regarding metrics, detail resolution and amplitude of susceptibilities in the evaluation on three different data sets with varying FoV orientation and voxel-size (Figure 4) on 1 mm isotropic data from Shi et al. (2022). The susceptibility maps computed with the adaptive model are the ones mostly similar to the ground truth, visually and in metrics. Differences in the reconstructed maps are present in the globus pallidus (Figure 4A, white arrows) and in the optic radiation (Figure 4A, white circled arrows). While both adaptive models underestimate the susceptibility in the globus pallidus, an overestimation of susceptibilities is visible for the conventional U-Net and AFTER-QSM. The susceptibility map computed by AFTER-QSM (Figure 4A) exhibits a generally increased contrast level. As indicated by the difference maps, the adaptive U-Net and AFTER-QSM delineated most accurately the substantia nigra and red nuclei (Figure 4B, white circled arrows) on the tilted data set, whereas the susceptibility map of the conventional U-Net did not show a clear boundary between these structures. Similar findings are observed on the anisotropic data set, where all models have difficulties in delineating the substantia nigra and red nuclei (Figure 4C, white circled arrows), however, AFTER-QSM achieves the clearest delineation. The adaptive model without transfer learning showed visually the largest deviations to the COSMOS ground truth data set. AFTER-QSM and the adaptive model without transfer learning achieved the lowest image metrics. All visual findings are supported by the respective difference maps. The NRMSE deviates between the models in a greater range as the SSIM.

**Figure 5:**
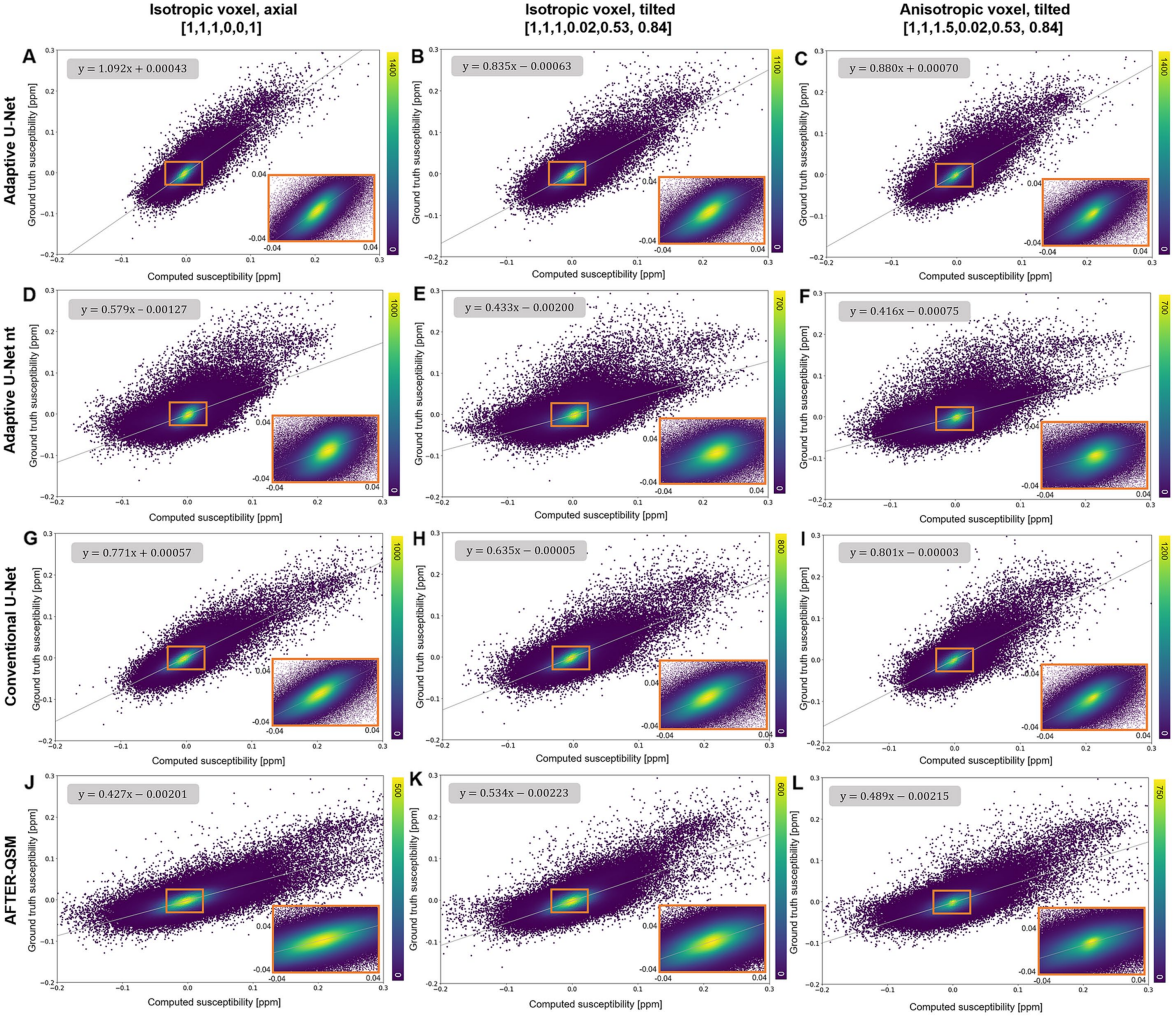
Scatter plots of reconstructed susceptibilities across the whole brain with respect to the ground truth COSMOS susceptibility on different brain data sets. The scatter plots for the data points sampled from susceptibility maps computed with the adaptive U-Net with transfer learning **(A–C)**, the adaptive U-Net without transfer learning **(D–F)**, the conventional U-Net with transfer learning **(G–I)** and AFTER-QSM **(J–L)** are shown from left to right, respectively. Scatter plots generated from data sets with 1 mm^3^ isotropic voxels and pure axial FoV alignment ($\vec{o}=$[0, 0, 1]^T^, **A,D,G,J**), with 1 mm^3^ isotropic voxels and tilted FoV ($\vec{o}=$[0.02, 0.53, 0.84]^T^, **B,E,H,K**), and with anisotropic voxels (1 mm × 1 mm × 1.5 mm) and tilted FoV $(\vec{o}=$[0.02, 0.53, 0.84]^T^, **C,F,I,L**) are shown from top to bottom, respectively. The density of data points is encoded in color. The fitting curve was obtained by least-squares fitting. Enlarged sections are framed by an orange rectangle.

The scatter plots of randomly drawn susceptibilities (Figure 5) from the maps computed from *in-vivo* brain data (Figure 4) revealed a point cloud centered on a straight line for all models. On the isotropic and non-tilted data set (Figure 5A), the slope of 1.092 from the fitting line of the adaptive U-Net marks the closest agreement between the computed and ground truth COSMOS susceptibilities. The same model revealed its lowest slope (0.835) on the isotropic tilted data set (Figure 5B). The fitted slopes of the data reconstructed with the conventional U-Net varied between 0.635 and 0.801 (Figures 5G–I). The susceptibility maps reconstructed with the adaptive U-Net without transfer learning produced the broadest point distributions and slopes between 0.416 and 0.579 (Figures 5D–F). For AFTER-QSM (Figures 5J–L), the point distributions are more closely centered on the fitting line compared to those of the adaptive model without transfer learning. The slopes of the fitting line ranged from 0.427 to 0.534.

**Figure 6:**
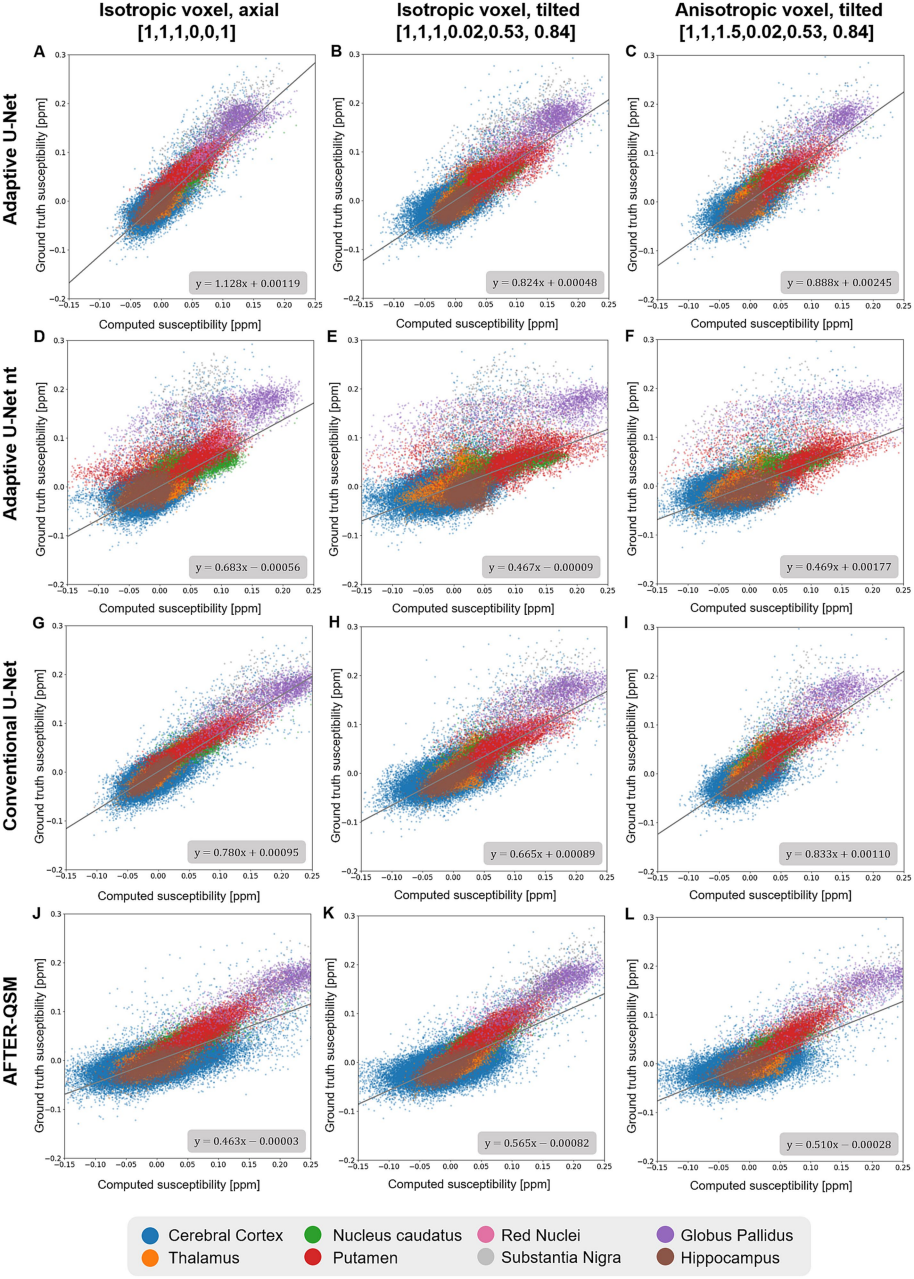
Scatter plots of deep gray matter regions of reconstructed susceptibility maps with respect to the ground truth COSMOS susceptibility on different brain data sets. The scatter plots in deep gray matter regions computed by the adaptive U-Net with transfer learning **(A–C)**, the adaptive U-Net without transfer learning **(D–F)** the conventional U-Net with transfer learning **(G–I)** and AFTER-QSM **(J–L)** are shown from left to right, respectively. Scatter plots generated from data sets with 1 mm^3^ isotropic voxels and pure axial FoV alignment ($\vec{o}=$[0, 0, 1]^T^, **A,D,G,J**), with 1 mm^3^ isotropic voxels and tilted FoV ($\vec{o}=$[0.02, 0.53, 0.84]^T^, **B,E,H,K**), and with anisotropic voxels (1 mm × 1 mm × 1.5 mm) and tilted FoV $(\vec{o}=$[0.02, 0.53, 0.84]^T^, **C,F,I,L**) are shown from top to bottom, respectively. The different deep gray matter regions are color-encoded. The fitting curve was obtained by least-squares fitting.

Scatter plots of susceptibilities within various gray matter regions (Figure 6) obtained from *in-vivo* brain data (Figure 4) revealed similar slopes like the ones determined across the whole brain (Figure 5). Agreement between the computed and ground truth susceptibilities was closest for the adaptive model as indicated by slopes varying between 0.824 and 1.128 (Figures 6A–C). The point distributions of the adaptive model without transfer learning (Figures 6D–F) and AFTER-QSM (Figures 6J–L) exhibit the largest dispersion, with fitting line slopes of about 0.5 being the lowest.

**Figure 7:**
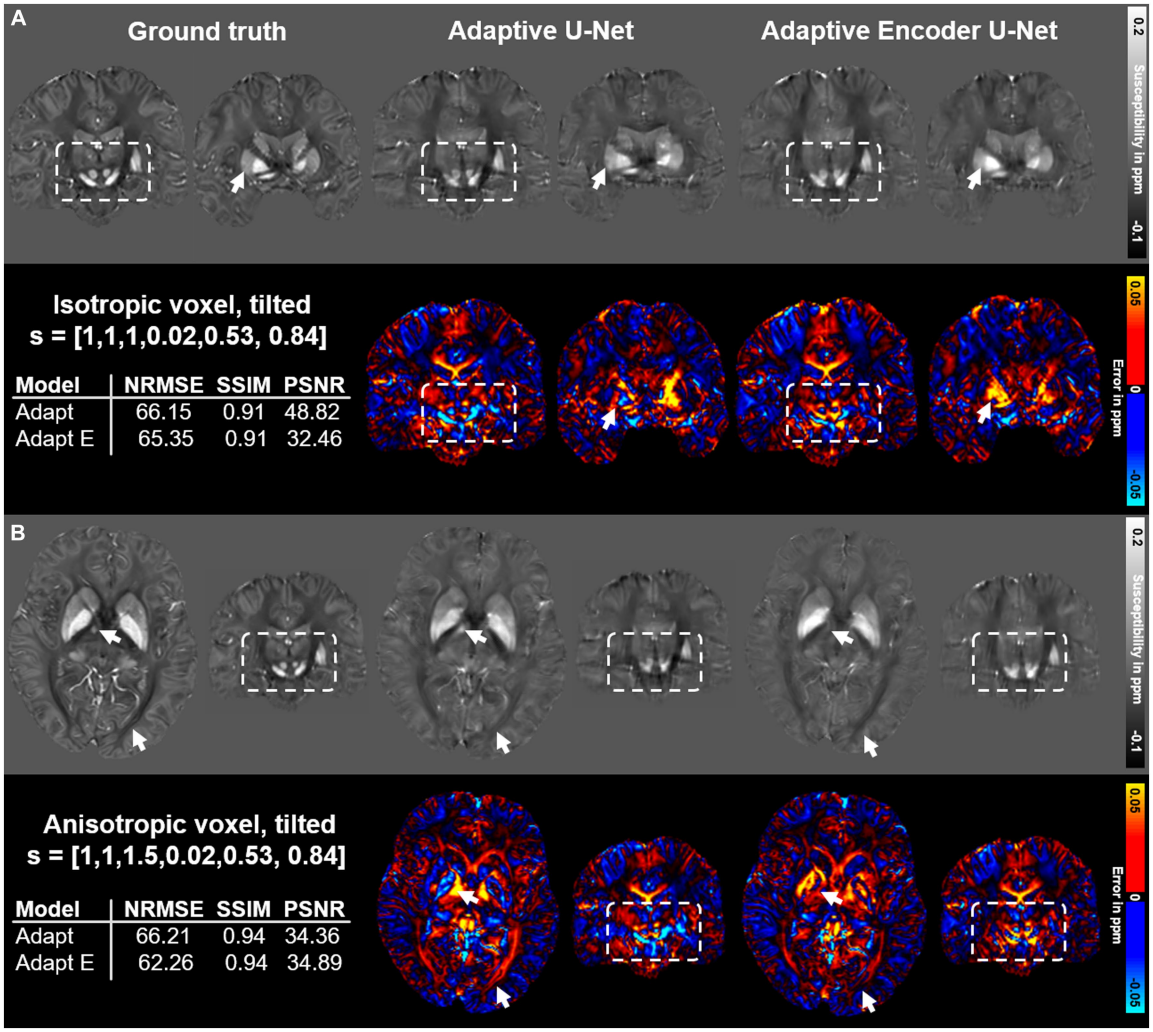
Comparison of the single adaptive layer model and the multiple adaptive layer model on brain data sets provided by Shi et al. (2022). Susceptibility maps were computed for two data sets by the adaptive U-Net with a single adaptive layer (Adaptive U-Net) and the adaptive encoder U-Net with the adaptive layer included from the first layer throughout the entire encoder (Adaptive Encoder U-Net). Both models experienced transfer learning. The two network models were evaluated on a dataset with isotropic voxel size (1 mm × 1 mm × 1 mm) and a tilted FoV ($\vec{o}=$[0.02, 0.53, 0.84]^T^) **(A)** and a dataset with anisotropic voxel size (1 mm × 1 mm × 1.5 mm) obtained by trilinear interpolation and a tilted FoV $(\vec{o}=$[0.02, 0.53, 0.84]^T^) **(B)**. The rows with gray background show the computed susceptibility maps, the rows with black background the respective difference maps to the ground truth. Arrows highlight prominent differences in the computed susceptibility maps. The normalized root mean squared error (NRMSE), the structural similarity index (SSIM) and the peak signal-to-noise ratio (PSNR) serve as quantitative image metrics and are presented left. Arrows and rectangles highlight prominent differences in the computed susceptibility maps. Transfer learning was performed on similar data from Shi et al. (2022).

The performance of two different configurations of the adaptive U-Net differing in the number of included adaptive layers based on two different brain data sets is summarized in Figure 7. The adaptive U-Net with a single adaptive layer in the first encoding block (Adaptive U-Net, Figure 1C) and the adaptive encoder U-Net with multiple adaptive layers (in total 10) included from the first convolution block throughout the entire encoder (Adaptive Encoder U-Net, Supplementary Figure S1) compute susceptibility maps of comparable image quality. The NRMSE decreased slightly from 66.15 to 65.35 on the tilted isotropic data set (Figure 7A) and from 66.21 to 62.26 on the tilted anisotropic data set (Figure 7B). SSIM values of the maps computed with the different models were equal. The difference maps of the adaptive encoder U-Net revealed higher susceptibilities in various regions with high iron content than the single layer adaptive U-Net, e.g., in the right putamen, globus pallidus, and substantia nigra (see arrows and rectangles in Figure 7A) and lower susceptibilities in white matter fiber tracts, e.g., pyramidal tracts, (see white rectangles in Figure 7B). The adaptive encoder U-Net achieves similar demarcation of brain structures like the substantia nigra and red nuclei (Figure 7, white rectangles).

**Figure 8:**
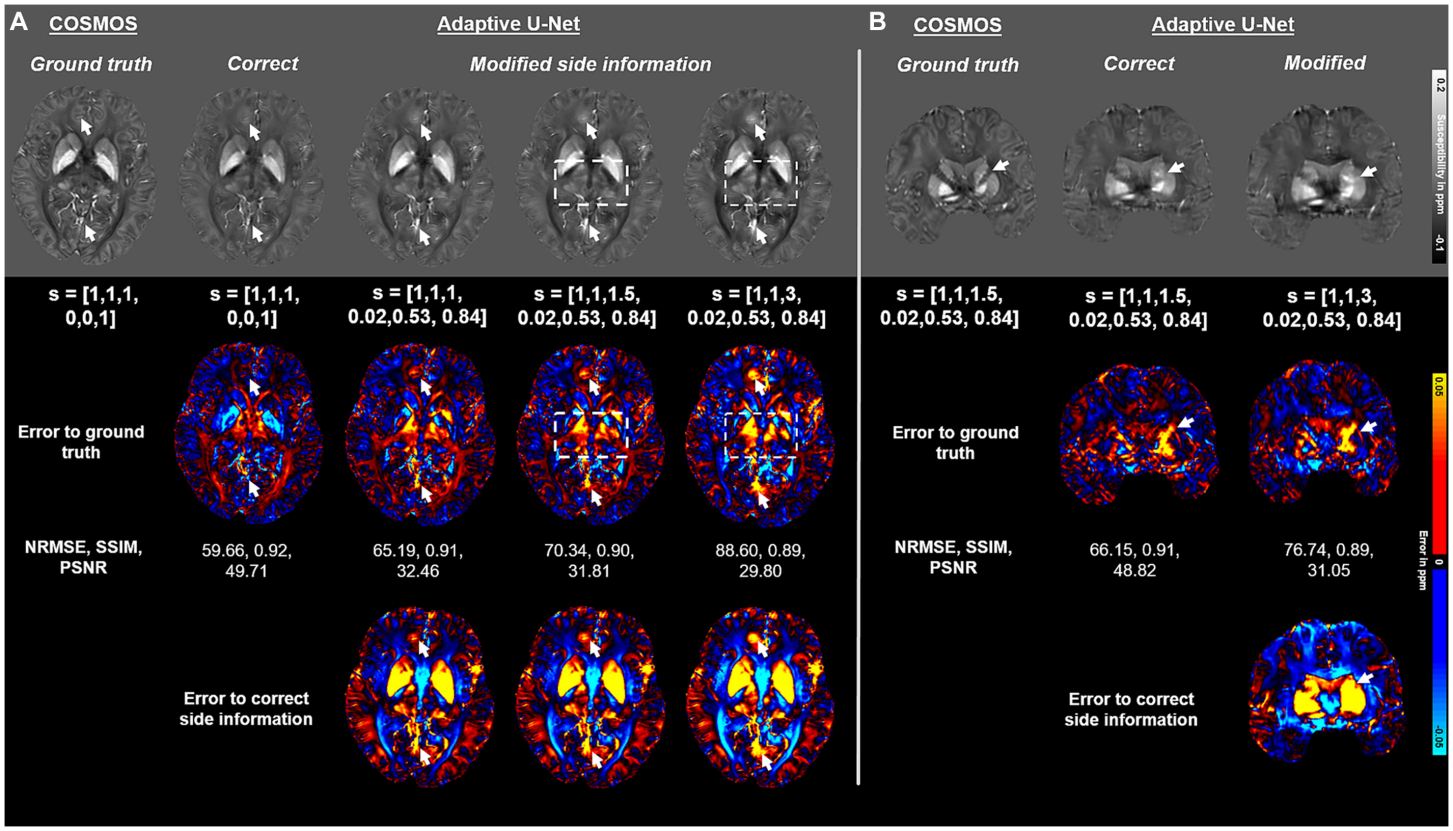
Impact of the side information array on susceptibility map reconstruction. The adaptive convolution layer of the adaptive U-Net was evaluated regarding the impact of the presented side information s on a brain dataset with 1 mm^3^ isotropic voxel size and pure axial FoV orientation ($\vec{o}=$[0, 0, 1]^T^) **(A)** and a dataset with anisotropic voxel size (1 mm × 1 mm × 1.5 mm) obtained by trilinear interpolation and a tilted FoV $(\vec{o}=$[0.02, 0.53, 0.84]^T^) **(B)**. The COSMOS ground truth map is presented in the first column, followed by the reconstructed susceptibility maps of the adaptive U-Net with correct and false side information arrays. The rows with black background show the respective difference map to the ground truth (second row of images) and the difference map to the susceptibility map reconstructed with the correct side information arrays (third row of images). Arrows and rectangles highlight prominent differences in the computed susceptibility maps. The normalized root mean squared error (NRMSE), the structural similarity index (SSIM) and the peak signal-to-noise ratio (PSNR) serve as quantitative image metrics and are presented left. Data provided by Shi et al. (2022).

The impact of the side information array s on the adaptive U-Net is presented in Figure 8. If the side information is modified to values that do not match the used acquisition parameters, the image quality deteriorates and deviations to the ground truth increase for both, myelin- and iron-containing structures. The reconstructions with false side information yielded higher NRMSE and lower SSIM compared to maps reconstructed with the correct one (Figure 8). As highlighted by the error maps, the susceptibility maps deviate from the ground truth the more the side information differs from the correct one. The arrows and the rectangles indicate structures that substantially deviate from the susceptibility maps computed with the correct side information.

**Figure 9:**
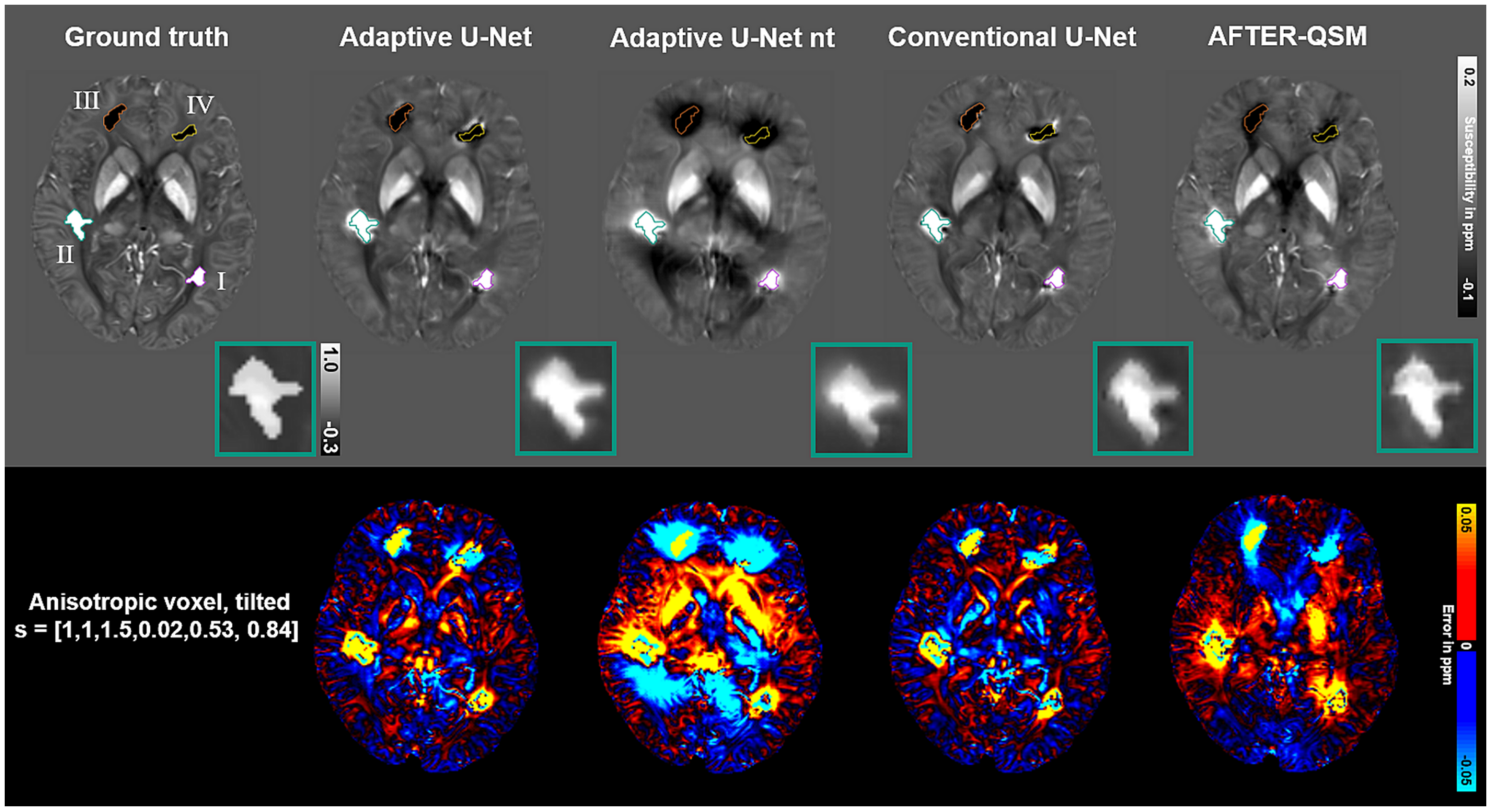
Evaluation of different network models on a simulated lesion data set. Susceptibility maps computed by the Adaptive U-Net with transfer learning (Adaptive U-Net), the adaptive U-Net without transfer learning (Adaptive U-Net nt), the conventional U-Net (Conventional U-Net) and AFTER-QSM are presented from left to right, respectively. The different approaches were evaluated on a data set from Shi et al. (2022) with anisotropic voxel size (1 mm × 1 mm × 1.5 mm) obtained by trilinear interpolation and a tilted FoV $(\vec{o}=$[0.02, 0.53, 0.84]^T^). The different lesions are numbered with roman numerals and color-encoded. The turquoise rectangle shows a close-up of lesion II with a given average susceptibility of 0.72 ± 0.31 ppm. The difference maps with respect to the COSMOS ground truth are shown in rows with black background.

Susceptibility maps reconstructed from a data set with pathological lesions using different field-to-susceptibility models are presented in Figure 9. The overall susceptibility of the adaptive U-Net was closest to the COSMOS ground truth map, particularly in deep gray matter regions. The appearance of lesions were blurred across their original structural boundaries in all susceptibility maps, with the difference maps indicating largest deviations especially for lesion III (orange, top right hemisphere, susceptibilities of −0.68 ± 0.34 ppm). Lesion II (turquoise, susceptibilities of 0.72 ± 0.31 ppm) is shown at an enlarged scale, revealing subtle differences in the reconstructed lesion susceptibilities. The mean and standard deviations of lesion susceptibilities are summarized in Table 2, where the AFTER-QSM approach revealed closest agreement to the ground truth lesion susceptibilities. The adaptive U-Net had larger susceptibility deviations for lesions with negative susceptibility differences (e.g., calcified lesion III, IV) than for the ones with positive susceptibility differences (e.g., hemorrhagic lesion I, II).

**Table 2:** Magnetic susceptibility values (mean values ± standard deviations) in ppm measured on susceptibility maps reconstructed from simulated lesion data set using different field-to-susceptibility inversions methods.

Region	Ground truth	Adaptive	Adaptive nt	Conventional	AFTER-QSM
*I, Purple*	0.38 ± 0.16	0.42 ± 0.18	0.30 ± 0.15	0.35 ± 0.14	0.40 ± 0.17
*II, Turquoise*	0.72 ± 0.31	0.80 ± 0.38	0.62 ± 0.29	0.66 ± 0.30	0.69 ± 0.33
*III, Orange*	−0.68 ± 0.34	−0.39 ± 0.17	−0.52 ± 0.20	−0.36 ± 0.21	−0.66 ± 0.30
*IV, Yellow*	−0.38 ± 0.15	−0.23 ± 0.09	−0.34 ± 0.10	−0.22 ± 0.11	−0.41 ± 0.18

### 3.4 *In-vivo* brain measurements

**Figure 10:**
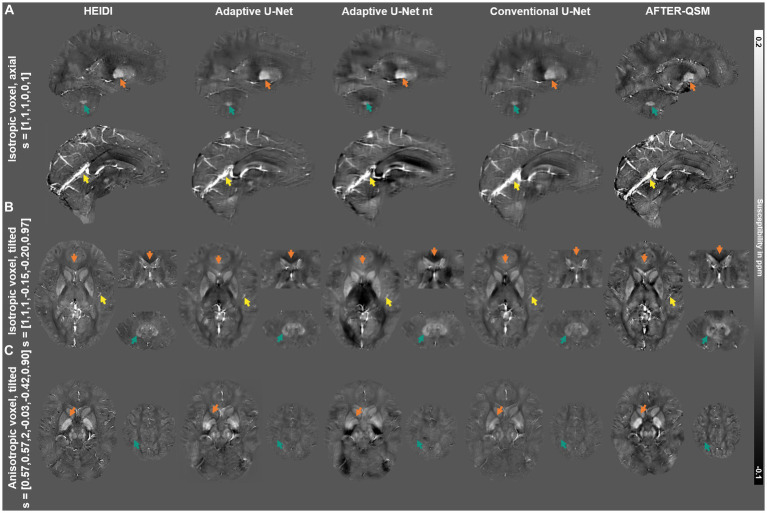
Comparison of the different network models on *in vivo* brain data sets acquired within this study. Susceptibility maps computed with HEIDI (Schweser et al., 2012), the adaptive U-Net with transfer learning, the adaptive U-Net without transfer learning, the conventional U-Net with transfer learning and AFTER-QSM are presented from left to right, respectively. The different QSM approaches were evaluated on a dataset with 1 mm^3^ isotropic voxel size and pure axial acquisition ($\vec{o}=$[0, 0, 1]^T^) **(A)**, a dataset with 1 mm^3^ isotropic voxel size and a 12-degree tilted FoV ($\vec{o}=$[–0.15, −0.20, 0.97]^T^) **(B)** and a dataset with anisotropic voxel size (0.57 mm x 0.57 mm x 2 mm) and a 25-degree tilted FoV ($\vec{o}=$[–0.03, −0.42, 0.90]^T^) **(C)**. Arrows highlight prominent differences in the computed susceptibility maps.

The susceptibility maps from HEIDI and the adaptive U-Net with transfer learning visually have closest agreement in computed susceptibilities on the isotropic non-tilted data set (Figure 10A) and in iron-laden structures such as the putamen (Figure 10A orange arrows) and the dentate nuclei (Figures 10A,B turquoise arrows). Furthermore, the adaptive U-Net and AFTER-QSM achieved improved delineation of large (Figure 10A yellow arrows) and small brain vessels (Figure 10B yellow arrows) than the conventional U-Net. Substantial differences in susceptibilities are also present in the genu of the corpus callosum (Figure 10B orange arrows), with the conventional U-Net reconstructing positive rather than negative susceptibilities. Although the high-resolution dataset (Figure 10C) is out-of-distribution data, since the voxel size of the scan was not part of the network training and hence, not on the learned filter manifold, the contrast between the susceptibility map from HEIDI and the adaptive U-Net are comparable, while less contrast is visible in the susceptibility map from the conventional U-Net. A shadow effect is visible in the vicinity of large veins in the cerebellum of the reconstructed susceptibility maps of the adaptive U-Net that is more pronounced in the map of the adaptive U-Net without transfer learning and less visible in the conventional U-Net. The reason for the shadowing may be attributed to the fact that the local field distribution is sparsely sampled across the slice encoding direction because of the high voxel aspect ratio (voxel size in slice encoding direction divided by the voxel size in-plane) of 3.5. The AFTER-QSM map additionally yields large scale heterogeneities in the cerebellum. All susceptibility maps from the adaptive U-Net without transfer learning substantially deviate from the HEIDI susceptibility and the adaptive U-Net and are prone to artefacts. Like in Figure 4, the susceptibility maps of AFTER-QSM revealed a higher contrast, a more specifically-larger co-domain than those of the adaptive U-Net and HEIDI, especially for deep gray matter regions in the high-resolution data (Figure 10C).

## 4 Discussion

For the first time, *a priori* information of voxel-size and orientation was successfully included into deep learning models via adaptive convolution for solving the ill-posed field-to-susceptibility problem. In addition, we demonstrated that pre-training on synthetic data and transfer learning to *in vivo* brain data is possible and substantially improves the reconstruction outcomes on *in vivo* data.

The evaluation across the different synthetic (Figure 3) and human brain data sets (Figures 4, 9, 10) revealed closest agreement between ground truth data and susceptibility maps computed by the proposed adaptive model. Due to the inclusion of Gaussian noise in network training, the U-Net-based approaches produce images with less noise than the susceptibility maps obtained by iterative reconstruction with HEIDI (Figure 10). The adaptive model achieves a nearly identical mapping of the susceptibility values on synthetic data (Figure 3C) and substantially lower deviations than the conventional model and AFTER-QSM on brain data (Figures 5, 6). The discrepancies of the conventional model in reconstructing the expected susceptibility range are most likely due to non-optimal optimization of its network parameters. In order to get as close as possible to the predicted range of susceptibility values, transfer learning of the network models to *in vivo* data is essential as indicated by the resulting data computed with the adaptive model with and without transfer learning (Figures 5, 6). In comparison to other deep learning approaches, the adaptive model achieved similar NRMSE and higher SSIM as the xQSM model (Gao et al., 2020) as reported by Shi et al. (2022) on their proposed data set. Our adaptive U-Net outperformed AFTER-QSM on *in-vivo* brain data (Figures 4–6) with substantially lower NRMSE and higher SSIM as well as slopes closer to 1. The results reported by Xiong et al. (2023) on different data sets from Shi et al. (2022), however, show PSNR similar to those of the adaptive model and slightly improved SSIM. AFTER-QSM is a deep learning approach that applies a U-Net for the dipole inversion problem and subsequently the REFINE network, a super-resolution network, to sharpen the image. Hence, the super-resolution approach and differences in training strategies – adaptive U-Net with pre-training on synthetic data and transfer learning on brain data and AFTER-QSM with direct training on *in vivo* brain data – are likely the explanation for differences in image sharpness on the measured brain data (Figure 10). Therefore, the integration of such a super-resolution technique into the adaptive model might further enrich the spatial details of the computed susceptibility maps. To test the robustness of the adaptive model against out-of-distribution data, a high-resolution MRI data set (voxel size: 0.57 mm × 0.57 mm × 2 mm) was considered. While the resulting susceptibility map of the adaptive U-Net exhibits a contrast similar to the one from HEIDI (Figure 10C), the map also shows difficulties in inverting the magnetic field. This is obvious close to large veins in the cerebellum, where the local field change is large but the local field is only sampled sparsely due to the large voxel dimension along the slice encoding direction. Consequently, partial voluming of the intravascular but also the extravascular field contributions occur, leading to inaccurate field-to-susceptibility inversion. Additionally, difficulties of the adaptive layer extrapolating the learned manifold (such side information was not part of the training data set) might impact susceptibility map computation. These difficulties, however, also shows that the FMN in the adaptive layer substantially influences the network model by using the side information to determine the filter weights. To alleviate the dependence on the individual components of the voxel size $v_{i}$, the use of the voxel aspect ratio as side information is conceivable.

The simulated calcified and hemorrhagic lesions introduced susceptibilities that were out of the learned susceptibility distribution (Figure 9; Table 2). The synthetic data sets used for network pre-training have a broad susceptibility distribution, thus explaining the lower deviations of the adaptive model without transfer learning to the ground truth. During transfer learning, the adaptive model tailors its weights more specifically to the data distribution of the provided data sets. While we attempted to keep the susceptibility scale invariance during the transfer learning by using a mixture of measured phase data and field forward convolution of COSMOS data sets, the scale invariance of the final adaptive model seems to be more limited. In addition, the data sets used for transfer learning were from healthy subjects and, thus, not equipped with pathological lesions as well as with susceptibilities exceeding ±0.4 ppm. This might explain the larger deviations measured in lesions on susceptibility maps of the adaptive and conventional U-Net (Table 2, Lesion II, turquoise). Augmenting data sets with tissue pathologies in the transfer learning might alleviate this effect.

As comprehensively outlined in the methods section, we attempted to reduce the discrepancy between simulated data and *in vivo* data in multiple ways, for instance, less straight and sharp edges as well as Gaussian noise corruption. We also applied a transfer learning strategy to fine-tune the models’ network parameters towards brain data, which resulted in improved metrics and greater visual similarity to the ground truth map (Figure 4). Accordingly, the fine-tuned adaptive model notably increased the quality of computed susceptibility maps. In comparison to Figure 4, the calculated susceptibility maps from our MRI experiments were slightly deteriorated (Figure 10) due to the fact that data from our MRI machine was not seen by the network in the transfer learning procedure. The QSM processing pipeline and the MRI scanner itself are the two main contributors, affecting the local magnetic field distribution that served as input to our network models (QSM Consensus Organization Committee et al., 2024). The choice of the multi-channel coil combination algorithm, the phase unwrapping approach (Robinson et al., 2017) and the background field removal method (Schweser et al., 2017) impact the local magnetic field. Hence, slight variations, e.g., Laplacian-based phase unwrapping or best path phase unwrapping, might manifest for deep learning models in inconsistencies in the computed susceptibility map. Intrinsic scanner specific variations due to the manufacturer or even scanner model (Stamoulou et al., 2022) or the use of different MRI coils (Panman et al., 2019) additionally affect the data, leading to difficulties for deep learning approaches when applied to data from different installations (Yan et al., 2020).

The model architecture as well as training configuration also affect the performance of the network model. Our loss measure used for training (Eq. 4) was composed of two L_2_-Norms and thus optimizes towards intensity variations. The overall contributions of sharp edges to the loss are minor, hence, the intensity-based loss is low even if certain amounts of edges are missed. Since the number of edges in the synthetic data sets was generally higher as compared to those of *in vivo* data, we found that achieving SSIM metrics higher than 0.7 on synthetic data is sufficient to fine-tune the model towards *in vivo* data. In the task of single image super resolution of 2D photographic images, utilization of an edge-based loss function revealed improvements over the mean squared error loss (Seif and Androutsos, 2018). Hence, the inclusion of such an edge-based component might improve edge reconstruction in susceptibility maps as well. The tuning of hyperparameters for training the adaptive model as well as finding the optimal configuration of the FMN and the total number of the adaptive convolution layers was accomplished based on already published parameters that were adjusted iteratively towards better performance as indicated by Eq. 4. The first two convolutional layers of the standard U-Net primarily extract edge-components in their feature maps, which are directly influenced by changes in the voxel size and image orientation. As a result, we positioned the adaptive layer directly after these two convolution operations at the encoder (Figure 1C). Here, the relationship between the side information array and the changes in the image associated with this specific side information is more apparent, allowing the model to identify and learn the mapping more easily. The adaptive U-Net’s property to adapt to side information is also clearly illustrated in Figure 8, where we deliberately modified the side information (e.g., to higher slice thickness), while keeping the input local field consistent. The higher slice thickness (up to 3 mm) covers a larger tissue stack resulting in increased contrast to noise and partial voluming but also in a sparser sampling of the local field that needs to be accounted for by the deconvolution kernel in the inversion process (Eq. 2). The higher magnitude of variations to the ground truth with increasing slice thickness along with contrast changes in thalamic and frontal brain regions (Figure 8, arrows and rectangles), shows that the FMN and thus the adaptive layer have learned fundamental MRI dependencies. This indicates the importance of presenting the correct side information to the adaptive U-Net.

In addition to the adaptive U-Net with a single adaptive convolution layer, we also probed the inclusion of multiple adaptive convolution layers in the encoder (Figure 7; Supplementary Figure S1). While the NRMSE of the susceptibility maps reconstructed using the adaptive encoder U-Net improved slightly, the overall visual impression and SSIM remained identical in comparison to the adaptive U-Net with a single adaptive convolution layer. Additionally, the tremendous increase in network parameters from approximately $8.3\cdot 10^{6}$ to $683.9\cdot 10^{6}$ doubled the network training time from 25 h to 50 h and substantially increased the GPU memory cost from 26 GB to 43 GB. In deeper network layers, the feature maps encode abstract and complex high-level features that might have a minor direct correlation with the side information. Overall, as a trade-off, we integrated a single adaptive layer at the most effective stage in the network model, since this configuration yielded comparable outcome to ten adaptive layers, suggesting that passing additional information to deeper network layers may have limited benefits, and requires substantially lower computation demands.

Due to the complexity of the network and its tailoring to 3D data, automated hyperparameter search was not conducted. Our hyperparameter adjustments, for instance, included analyses on variations in learning rate, learning rate schedules, batch size and activation functions (Graf et al., 2023). With adaptive convolution, our goal was to integrate additional information in the network model, based on which specific network parameters are generated. Its proper implementation and its influence on convolution filter kernels, as well as activations maps, could be demonstrated (Figure 2). Technically related to adaptive convolution are dynamic convolutions (Klein et al., 2015) and dynamic filter networks (Jia et al., 2016). However, these two approaches generate network parameters based on the image itself and not based on additional information. The weight prediction approach of Meta QSM (Liu and Koch, 2019) is technically closely related to manifold learning and our proposed adaptive convolution approach. However, it differs in terms of fundamental assumptions and technical implementation. Furthermore, we consider the voxel-size and FoV-orientation, while Meta-QSM solely focuses on the image resolution. The goal of adaptive convolution is to achieve resolution and orientation invariance by presenting the additional information at crucial points in the network model, where it substantially benefits the model to learn the mapping between additional information and information-related changes in the image, while also maintaining the flexibility of traditional DL in terms of parameter optimization. However, Meta-QSM replaces all convolution layers with weight prediction layers in the network model, thereby greatly increasing the total number of network parameters and computational demands. The implementation of Meta-QSM also utilizes dilated weight prediction convolution layers in the bottleneck of the network and the ReLU activation function in general.

Future work will focus on fine-tuning adaptive convolution and synthetic data sets. To reduce the gap between simulated and real-world data, contributions not arising from isotropic susceptibility should be considered, including anisotropic susceptibility and microstructural effects (Wharton and Bowtell, 2015) as well as phase inconsistencies due to flow artifacts (Bilgic et al., 2021). For the application of deep learning models to other body parts than the brain, chemical shift and motion artifacts need to be considered as well (Hanspach et al., 2022). With these we aim to build a comprehensive pipeline to generate synthetic data for general network pre-training allowing the models to be fine-tuned to various body regions. Lastly, we propose further fine-tuning for a small number of epochs to specific in-house data, addressing scanner-specific attributes, to further improve susceptibility map computation.

## 5 Conclusion

We demonstrated the incorporation of *a-priori* information of acquisition parameters via adaptive convolution and the feasibility of transfer learning from synthetic to *in vivo* MRI data for solving the ill-posed field-to-susceptibility inversion for the first time. Conventional field-to-source inversion algorithms include additional parameters to find optimal solutions. Similarly, we are confident that providing *a-priori* information acts as valuable constraint for parameter optimization, guiding the network model towards more valid solutions. Training on synthetic data followed by transfer learning, seems to be a valid approach to address data scarcity of ground-truth QSM data for supervised learning approaches.

## Data availability statement

The datasets presented in this article are not readily available because the authors have no permission to share the 7 T data used for transfer learning. 3 T training data is publicly available. Other data sets are available upon request. The source code is available on GitHub (https://github.com/HMRICF/DL-QSM). Requests to access the datasets should be directed to SG, simon.graf@uk-halle.de.

## Ethics statement

The studies involving humans were approved by the Ethics Commission of the Medical Faculty, Martin-Luther-University Halle-Wittenberg. The studies were conducted in accordance with the local legislation and institutional requirements. The participants provided their written informed consent to participate in this study.

## Author contributions

SG: Conceptualization, Data curation, Formal analysis, Investigation, Methodology, Software, Validation, Visualization, Writing – original draft, Writing – review & editing. WW: Funding acquisition, Project administration, Resources, Writing – review & editing. AD: Conceptualization, Data curation, Funding acquisition, Project administration, Resources, Software, Supervision, Writing – review & editing, Writing – original draft.
