# Supplementary figures and images for: Incorporating a-priori information in deep learning models for quantitative susceptibility mapping via adaptive convolution
Source: Front Neurosci. 2024 Mar 11;18:1366165. doi: 10.3389/fnins.2024.1366165 (PMC10962327; doi:10.3389/fnins.2024.1366165)

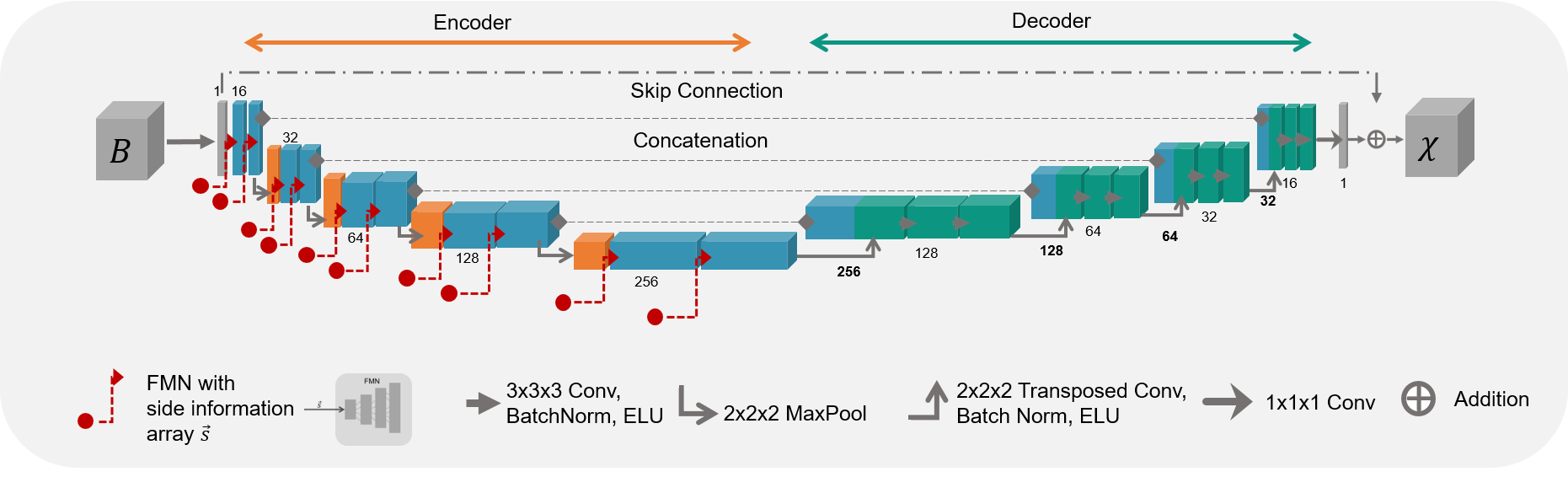

Supplement: Supplementary file 1 [file Image_1.TIF]
